# Gastrointestinal organoids: a next-generation tool for modeling human development

**DOI:** 10.1152/ajpgi.00199.2020

**Published:** 2020-07-13

**Authors:** Akaljot Singh, Holly M. Poling, Jason R. Spence, James M. Wells, Michael A. Helmrath

**Affiliations:** ^1^Division of General and Thoracic Surgery, Cincinnati Children’s Hospital Medical Center, Cincinnati, Ohio; ^2^Division of Developmental Biology, Cincinnati Children’s Hospital Medical Center, Cincinnati, Ohio; ^3^Department of Internal Medicine, Gastroenterology, University of Michigan Medical School, Ann Arbor, Michigan; ^4^Department of Cell and Developmental Biology, University of Michigan Medical School, Ann Arbor, Michigan; ^5^Department of Biomedical Engineering, University of Michigan College of Engineering, Ann Arbor, Michigan

**Keywords:** disease modeling, enteroid, gastrointestinal development, human intestinal organoid, stem cells

## Abstract

Gastrointestinal organoids are an exciting new tool for modeling human development, physiology, and disease in human tissue. Derived from pluripotent stem cells, gastrointestinal organoids consist of epithelial and mesenchymal cells organized in an intricate, three-dimensional structure that recapitulates the physiology and microscopic anatomy of the human gastrointestinal (GI) tract. In vitro derivation of gastrointestinal organoids from definitive endoderm has permitted an exploration of the complex signaling pathways required for the initial maturation of each individual gastrointestinal organ. Further maturation beyond an early fetal state currently requires transplantation into an immunocompromised host. Transplantation-induced maturation provides an opportunity to functionally interrogate the key mechanisms underlying development of the human GI tract. Gastrointestinal organoids can also be used to model human diseases and ultimately may serve as the basis for developing novel, personalized therapies for human intestinal diseases.

## Graphical abstract

**Figure:**
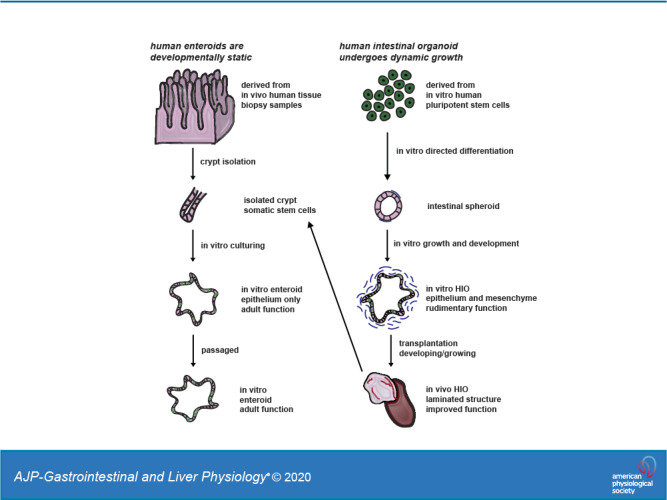

## INTRODUCTION

Remarkable insight into the key pathways underlying intestinal development, physiology, and diseases has been obtained using a variety of model organisms (33, 38, 50). Knowledge gained from animal models is not always directly applicable to human biology, as there are key differences between murine and human intestinal development and disease. For example, intestinal crypts develop postnatally in mice (6), but they develop during the first trimester in humans (15). Similarly, many animal models of human diseases have limited translational applications because they do not faithfully recapitulate all aspects of human disease phenotypes (17). It has been difficult to study gastrointestinal viral infections, for instance, such as norovirus, in murine models because the animals are not susceptible to human viruses (12). Thus, it is vital to develop tools that model human development, physiology, and disease in human tissue, as understanding these mechanisms will be instrumental to developing novel therapies for human intestinal disorders.

**Fig. 1.**
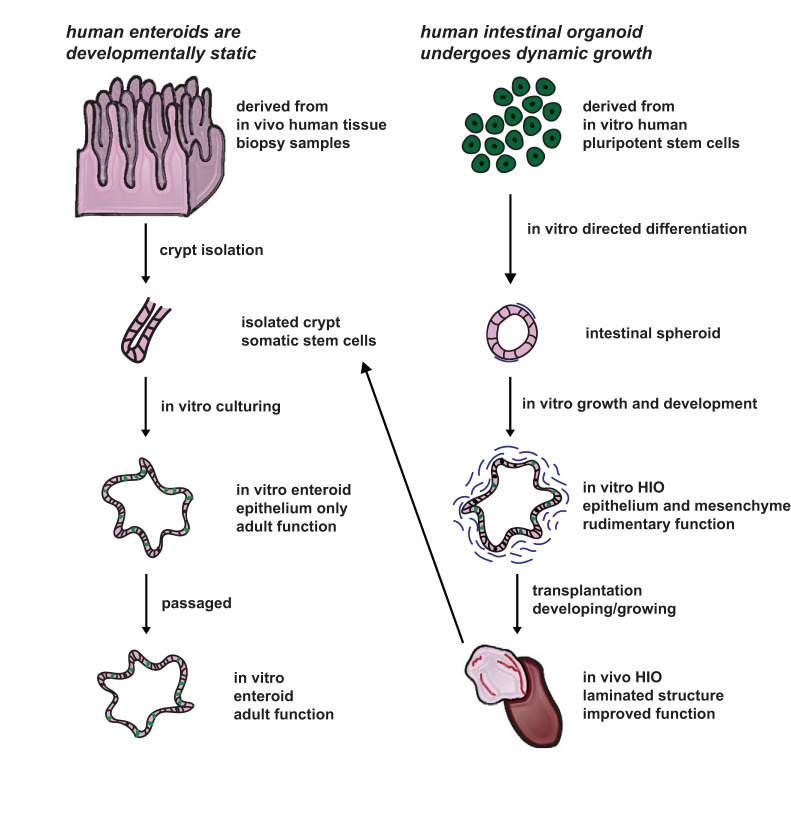
Human enteroids are developmentally static, whereas human intestinal organoids (HIOs) are developing dynamically. Enteroids are derived from a patient’s surgical or biopsy sample. Crypts are isolated from the tissue and, through in vitro culture, functional units of gut epithelium, or enteroids, grow. These structures may be passaged and exponentially expanded. HIOs are derived from pluripotent stem cells and, through directed differentiation, develop into functional units containing both epithelium and mesenchyme. Upon transplantation and engraftment, crypts may be isolated from HIO tissues to generate enteroids.

Within the last decade, two revolutionary tools have been developed for modeling human intestinal physiology and disease in humans: enteroids (32, 40) and human intestinal organoids (HIOs) (39) (Fig. 1). Enteroids, grown in vitro, are three-dimensional (3-D) intestinal epithelium-only structures that are derived from intestinal stem cells collected from patients (14, 31, 32). Enteroids mimic some aspects of the form and functionality of the donor tissue from which they are derived (14, 31, 32). For example, they display all of the differentiated epithelial cell types found in the human intestine, including enterocytes, goblet cells, Paneth cells, and enteroendocrine cells (32). Moreover, they possess the capacity to be cryopreserved and exponentially expanded (23) and can be derived from healthy or diseased epithelial tumors (32). Because they are derived from somatic tissue, they retain the epigenetic signatures of adult intestinal epithelium (20). Enteroids serve as a patient-specific tool for studying diseases that impact the intestinal epithelium, such as necrotizing entercolitis and cystic fibrosis (3, 9, 22). Additionally, enteroids have been used to study pathogenic interactions such as human norovirus and *Escherichia coli* with the intestinal epithelium (12, 16). Enteroids have been used to study the physiology of the adult intestinal crypt in both homeostatic and regenerative states (5). Although enteroids cultured in the traditional fashion are excellent tools to address in vitro questions regarding the intestinal epithelium, they lack mesenchymal, neuronal, and immune compartments. This can limit their use for studying complex, nonepithelial intestinal physiology and pathology, including diseases such as Hirschsprung disease, which impacts the enteric nervous system, and Berdon syndrome, which impacts the enteric smooth muscle (1, 4). More recently, efforts have been made to coculture enteroids with other intestinal components, including myofibroblasts (21). Another limitation of enteroid models is that they have an extremely low engraftment rate upon transplantation (48) and are therefore not an efficient tool for in vivo work. Thus, enteroids can only be used to address questions about in vitro physiology and pathology, potentially restricting their use for exploring questions involving complex cell-to-cell interactions. This includes autoimmune conditions, including celiac disease and ulcerative colitis. Finally, it is difficult to study intestinal development using enteroids, as only epigenetic developmental changes have been noted in enteroids derived from fetal human intestine. A complementary model is required to explore questions regarding complex cellular interactions, human intestinal development, and the physiology and pathology of the mesenchymal, neuronal, and immune compartments.

Unlike enteroids, human intestinal organoids are generated using directed differentiation of pluripotent stem cells (PSCs) (26, 39). This process involves mimicking key developmental stages by altering the growth factors that the PSCs are exposed to in a stepwise manner. HIOs possess the ability to self-organize, and contain both epithelial and mesenchymal cells arranged in a complex 3-D structure. In vitro, HIOs do not mature beyond an early fetal state (13, 30). Cellular and morphological maturation requires transplantation into an in vivo environment, such as an immunocompromised animal model. When HIOs are transplanted into the renal subcapsular space, they increase cellular complexity and become more structurally similar to the native intestine, with a crypt-villus axis and all layers of smooth muscle found in adult human tissue (46). Additionally, transplanted HIOs (tHIOs) develop intestinal vasculature through contribution from the host (46). As a base model, HIOs lack both immune cells and the enteric nervous system. However, later work has allowed for the inclusion of the enteric nervous system (47), and efforts to promote development of the intestinal immune system are currently underway. Thus, HIOs can be used to model human intestinal development and to study nonepithelial intestinal diseases. The most significant challenge for using HIOs to study human development and physiology is the need to surgically transplant HIOs into immunocompromised animal models to induce maturation, as the surgery is technically demanding and the maturation process is time consuming. Despite this limitation, HIOs are an excellent complementary model to enteroids.

In this review, we will discuss recent efforts to use PSC-derived gastrointestinal organoids to study human development. We also highlight unique applications of this technology for studying and developing potential therapies for human intestinal disease.

## IN VITRO MODELING OF GASTROINTESTINAL DEVELOPMENT

**Fig. 2.**
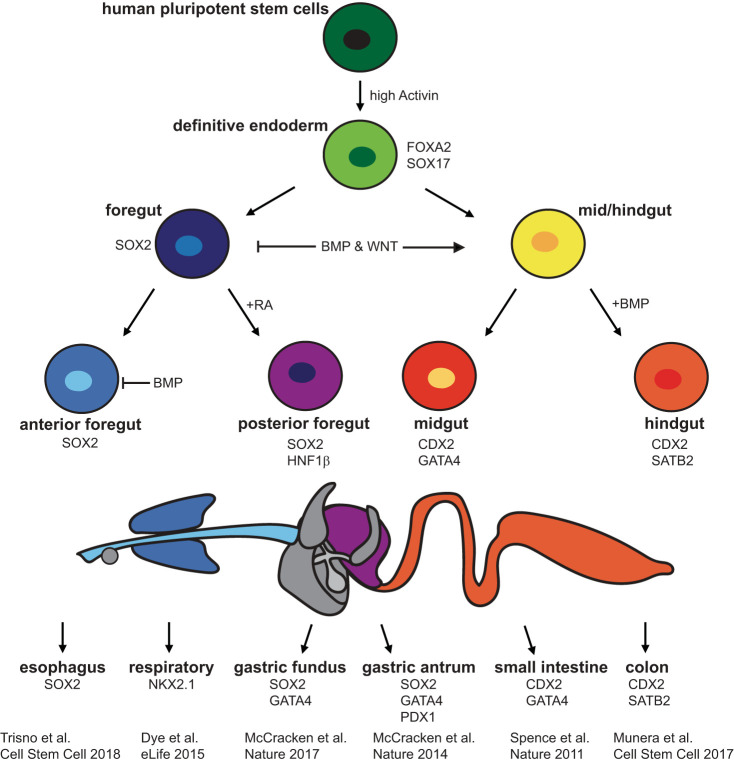
Differentiation pathway schematic for gastrointestinal organoids. Pluripotent stem cells exposed to high activin become definitive endoderm, which can then be driven toward foregut, or mid/hindgut fates through modulation of bone morphogenic protein (BMP) and WNT. From here, additional cues are provided to produce a variety of gastrointestinal organoids, including esophageal, respiratory, gastric, and intestinal. CDX2, caudal type homeobox 2; HNF1β, hepatocyte nuclear factor-1β; PDX1, pancreatic and duodenal homeobox 1; RA, retinoic acid; SATB2, special AT-rich sequence-binding protein 2; SOX2, SRY-box 2; SOX17, SRY-box 17.

The process of deriving gastrointestinal organoids from PSCs has provided researchers with an in vitro platform for studying the mechanisms underlying the development of the human gastrointestinal (GI) tract. By differentiating PSCs into gastrointestinal organoids based on the knowledge gained from Xenopus and other animal models, researchers can probe the temporospatial cues required for the differentiation of human GI organs. The first PSC-derived gastrointestinal organoid, the HIO, was developed by Spence et al. (39) in 2011. The authors found that exposing definitive endoderm (DE), 90% of which expressed the DE marker SRY-box 17 (SOX17) and 2% of which expressed the mesenchymal marker Brachyury, to fibroblast growth factor 4 (FGF4) and WNT3A for 96 h resulted in the formation of 3-D spheroids with both epithelial and mesenchymal components (39). These components displayed stable expression of caudal type homeobox 2 (CDX2), a key regulator of intestinal specification (39). As demonstrated in Fig. 2, researchers continue to study the temporospatial cues underlying human GI development. More recently, Tsai et al. (42) found that increasing exposure time of the spheroids to FGF4 and the small molecule GSK3β inhibitor, CHIR99021, which stabilizes β-catenin and activates WNT signaling, resulted in the development of HIOs that more closely resembled the ileum than the duodenum. Múnera et al. (28) found that exposing CDX2+ spheroids to bone morphogenic protein 2 (BMP2) for 3 days resulted in the stable expression of the colonic markers special AT-rich sequence-binding protein 2 (SATB2) and mucin 5B. Others have also been exploring the mechanisms underlying the development of anterior GI organs, including stomach and esophagus. McCracken et al. (25) found that exposing DE to noggin in addition to FGF4 and WNT3A, followed by a 24-h pulse of retinoic acid (RA) results in the creation of SRY-box 2 (SOX2)-positive and hepatocyte nuclear factor-1β (HNF1β)-positive posterior foregut spheroids. Exposing posterior foregut spheroids cultured in Matrigel to RA, noggin, and epidermal growth factor (EGF) for an additional 72 h and then culturing them in EGF media for an additional 28 days results in the production of SOX2-positive/pancreatic and duodenal homeobox 1 (PDX1)-positive human gastric antrum organoids (25). McCracken et al. (24) also found that adding CHIR99021 to the media of posterior foregut spheroids resulted in the formation of human fundic organoids, as indicated by expression of Iroquois-class homeodomain (IRX) proteins 2, 3, and 5 (24). Finally, Trisno et al. (41) found that by exposing DE to just a 1-day pulse of WNT3A instead of a 3-day pulse, they could generate anterior foregut spheroids. Exposing anterior foregut spheroids to a 1-day dose of RA followed by 65 days of supplementation of EGF resulted in the formation of human esophageal organoids (41). Manipulation of temporal exposure of DE to specific growth factors in vitro has thus provided researchers with a new tool for studying human gastrointestinal development.

In addition to permitting study of development of the organ as a whole, in vitro gastrointestinal organoids are an excellent system for studying the transcription factors and complex signaling pathways underlying differentiation of specific cell types present in the human gastrointestinal tract. For example, interrogation of enteroendocrine cell (EEC) lineage differentiation in human intestinal tissue has been carried out in HIOs. Knockdown of the transcription factor *ARX* resulted in 60–80% reduction in cholecystokinin, secretin, and preproglucagon mRNA, indicating that *ARX* is required for the development of specific EEC subtypes in human intestine (10). Moreover, through doxycycline-induced expression of neurogenin 3 (*NEUROG3*), Sinagoga et al. (37) increased the number of EECs in the intestinal epithelium from 1% to 25%, demonstrating that *NEUROG3* is sufficient for inducing development of EECs. This system allowed them to explore the kinetics of potential downstream targets of *NEUROG3* that had previously not been studied in either human or mouse intestine (37). They later applied this technology to exploring the impacts of specific, patient-derived *NEUROG3* mutations on development of EECs in both human intestine and human pancreas and found that the half-life of *NEUROG3* in the intestinal epithelium was half that of the half-life of *NEUROG3* in the pancreatic epithelium (49), explaining why many patients with *NEUROG3* mutations are able to maintain proper glycemic control. In vitro gastrointestinal organoids thus permit exploration of human development and disease at both the organ and cellular levels.

## IN VIVO MODELING OF HUMAN INTESTINAL DEVELOPMENT

Although in vitro gastrointestinal organoids serve as a platform for studying the early developmental pathways required for specification of the GI tract, they are not suited for studying development beyond the first trimester. This is because in vitro gastrointestinal organoids represent early fetal structures (13, 30) that cease maturation after 28–45 days in culture. To develop more mature tissue that was more suited for physiological studies, Watson et al. (46) transplanted in vitro HIOs that had been cultured for 28 days into the renal subcapsular space of immunocompromised mice. Six to eight weeks posttransplantation, the transplanted HIOs (tHIOs) had grown into intestinal structures that were up to 1 cm in diameter (46). Unlike in vitro HIOs, tHIOs developed a crypt-villus axis and organized mesenchyme, which included both layers of intestinal smooth muscle, indicating maturation of both epithelium and mesenchyme (46). tHIOs were also found to have all of the major differentiated epithelial cells, including goblet cells, Paneth cells, enterocytes, enteroendocrine cells, and stem cells (46). Analysis of graft functionality not only demonstrated the presence of all major lineages of EECs (37) and a differentiated brush border (46), but also demonstrated that the EECs were capable of secreting hormones in response to a glucose challenge (37) and that the enterocytes were capable of digesting and absorbing nutrients (46). Cortez et al. (8) replicated these findings through transplantation of HIOs into the mesentery, a more physiologically relevant engraftment site given its proximity to the intestine and its role in supporting intestinal development. RNA sequencing analysis has demonstrated that tHIOs harvested at 8 wk posttransplant more closely resemble late third trimester human fetal intestine (13, 30). Later studies with human colonic organoids (HCOs) also found that transplantation induced tissue maturation, as transplanted HCOs developed a substantial number of crypts (28). Thus, transplantation yields more mature tissue for physiological and developmental studies.

Transplantation-induced maturation raises the question of what allows HIOs to engraft and mature posttransplantation. Because enteroids do not readily transplant unless exposed to healthy mesenchyme (18, 21) and in vitro HIOs have a substantial mesenchymal component, it seems likely that mesenchymal populations are necessary for directing HIO engraftment and maturation posttransplantation. The fact that human gastrointestinal organoids with minimal mesenchymal support, including fundic, antrum, and esophageal organoids, do not engraft and develop into more mature structure posttransplantation further supports this hypothesis. Unfortunately, little is known about individual fibroblast populations, although some single-cell work defining these populations is currently underway (11, 29). Some work in mice has shown that PDGFRα+ mesenchymal cells help direct development of villi in developing murine intestine (45) and forkhead Box L1 (FOXL1+) fibroblasts maintain adult murine crypts at homeostasis (2, 34). However, recent work has demonstrated that human colonic fibroblasts do not share transcriptional profiles with murine colonic fibroblasts (19). There is thus a need to define human intestinal fibroblast populations in tHIOs and to functionally interrogate their roles in intestinal development, physiology, and pathology.

**Fig. 3.**
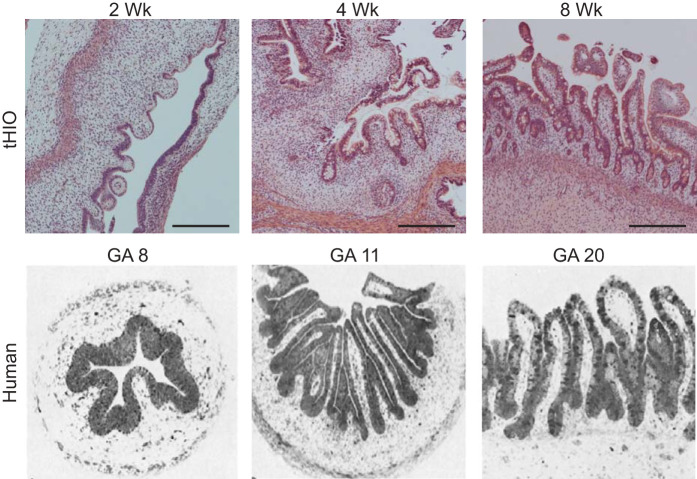
In vivo human intestinal organoid (HIO) development is similar to fetal human intestinal development. Hematoxylin and eosin-stained sections of transplanted HIOs (tHIOs) harvested 2, 4, and 8 wk posttransplantation (*top*) compared with historical sections of fetal human intestine (*bottom*). Development of epithelial structuration in tHIOs progresses in a similar fashion to native tissue. [Human intestine images are reproduced with permission (15).]

Another major question raised by transplantation-induced maturation is whether tHIOs can be used to model human fetal intestinal development. A recent time-course image analysis of tHIOs and comparison to images of human fetal intestine in the literature indicated (15) that the timeline of tHIO maturation mimics human fetal intestinal development. Specifically, villi begin to emerge after 2 wk in tHIOs and gestational week 8 in human intestine (15). Crypts develop at 4 wk in tHIOs and at gestational weeks 10–11 in human intestine (15) (Fig. 3). Thus, histologically, the pattern of tHIO development in vivo seems to replicate human fetal intestinal development. Further experimentation and benchmarking to primary tissue is required to understand the pathways underlying development of specific intestinal structures, including crypts, villi, and smooth muscle.

From a developmental perspective, tHIOs are also an exciting tool to study the development of intestinal complexity. However, HIOs lack all the components of adult human intestine, including an enteric nervous system (ENS), an immune system, an endogenous vasculature, and exposure to microbiota and luminal contents, including nutrients. These components are known to have an impact on the intestinal barrier function, gene expression, and development in murine models (7, 43, 44). Developing methods to individually provide these components to tHIOs will permit researchers to understand their impact on human intestinal development. Workman et al. (47) recently supplied an ENS to in vitro HIOs. They found that following transplantation of HIO-ENS cocultures, tHIOs with an ENS developed all the major neuronal subtypes found in the human intestine (47). These neurons were able to fire action potentials and induce muscle contraction (47). They further discovered that addition of an ENS had a profound impact on tHIO epithelium, including increased proliferation in the stem cell compartment and increased expression of stem cell genes, such as leucine-rich repeat-containing G protein-coupled receptor 5 (47). They also found alterations in EGF and TGF-β signaling as well as decreased expression of genes associated with secretory lineages, including goblet cells and Paneth cells (47). In addition to this, a greater percentage of the vasculature present in tHIOs with an ENS was human in origin as compared with the vasculature in control tHIOs (Poling HM, unpublished observations), suggesting that the ENS also plays a role in patterning the intestinal mesenchyme. Further work is required to tease out the ENS’s role in the development of intestinal fibroblasts and smooth muscle, as well as to provide an ENS to other gastrointestinal organoids.

Researchers also have used tHIOs to examine the impact of mechanical strain on the development of human intestine. Previously, it had been demonstrated in avian models that mechanical strain was important for both villification and localization of the stem cell compartment (35, 36). To determine what role mechanical strain played in development of human small intestine, Poling et al. (30) surgically implanted nitinol springs in tHIOs ten weeks posttransplant. Two weeks postsurgery, tHIOs with strain showed marked signs of maturation, including increased villus height and crypt depth (30). Thickness of both the longitudinal and circular muscle layers was also increased, a finding that was correlated to improvement in muscle function (30). Thus, mechanical strain appeared to induce maturation of both the epithelial and mesenchymal compartments. RNA sequencing analysis demonstrated that tHIOs with strain clustered more closely with infant small intestine than shams (30). Sequencing analysis also revealed upregulation of the MAPK/ERK, ERBB, and TBF-β signaling pathways as a result of strain (30), a result that was verified with protein-level expression (30). Ultimately, this study demonstrated that there was a positive impact of strain on the development of the tHIO. Future work will be required to further explore the mechanisms through which strain induces maturation of both the epithelial and mesenchymal compartments, as well as to determine whether these mechanisms can be used to further mature HIOs in vitro.

## CONCLUSION AND FUTURE DIRECTIONS

PSC-derived gastrointestinal organoids have proven to be an effective tool for studying the development of the human gastrointestinal tract. In vitro gastrointestinal organoids have been especially useful for delineating the key mechanisms underlying the specification of endoderm into different GI lineages, as well as for exploring the pathways required for the differentiation of specific cell types. In vivo transplantation models have been developed to further induce maturation beyond the first trimester. These models have been especially effective for studying the impact of components of the GI tract not present in vitro, such as strain. Understanding the pathways underlying transplantation-induced maturation will be instrumental for further differentiating organoids in vitro.

Future enhancement will involve increasing the complexity of organoid systems. Particularly, there is interest in providing organoids with a functional immune system, as the GI tract is constantly exposed to luminal antigens and microbes (27). Efforts to supply tHIOs with an immune system and to expose them to the microbes present in the murine GI tract are currently underway. Supplying tHIOs with these components will be instrumental for examining the interactions between the microbiome, the immune system, the epithelium, and the mesenchyme. Because the mesenchyme appears to be the component required for successful engraftment and maturation, efforts are currently underway to define individual mesenchymal populations in tHIOs, compare the cell types present in tHIOs to those present in the developing human intestine, and examine their impact on in vivo engraftment and maturation. Understanding these populations will be crucial for supplying other gastrointestinal organoids that currently lack substantial mesenchyme with the components required for engraftment. Ultimately, current efforts to bioengineer more complex organoids will be instrumental for creating personalized tissue to study human gastrointestinal development and disease.

## DISCLOSURES

No conflicts of interest, financial or otherwise, are declared by the authors.

## AUTHOR CONTRIBUTIONS

H.M.P. prepared figures; A.S. drafted manuscript; A.S., H.M.P., J.R.S., J.M.W., and M.A.H. edited and revised manuscript; A.S., H.M.P., J.R.S., and J.M.W. approved final version of manuscript.
